# The epidemiological characteristics of dengue in high-risk areas of China, 2013–2016

**DOI:** 10.1371/journal.pntd.0009970

**Published:** 2021-12-20

**Authors:** Shaowei Sang, Qiyong Liu, Xiaofang Guo, De Wu, Changwen Ke, Jing Liu-Helmersson, Jinyong Jiang, Yuwei Weng, Yiguan Wang

**Affiliations:** 1 Clinical Epidemiology Unit, Qilu Hospital of Shandong University, Jinan, Shandong, People’s Republic of China; 2 Clinical Research Center of Shandong University, Jinan, Shandong, People’s Republic of China; 3 Department of Epidemiology and Health Statistics, School of Public Health, Shandong University, Jinan, Shandong, People’s Republic of China; 4 State Key Laboratory of Infectious Disease Prevention and Control, Collaborative Innovation Center for Diagnosis and Treatment of Infectious Diseases, National Institute for Communicable Disease Control and Prevention, Chinese Center for Disease Control and Prevention, Changping, Beijing, People’s Republic of China; 5 Yunnan Provincial Center of Arborvirus Research, Yunnan Provincial Key Laboratory of Vector-borne Diseases Control and Research, Yunnan Institute of Parasitic Diseases, Pu’er, Yunnan, People’s Republic of China; 6 Institute of Microbiology, Guangdong Provincial Center for Disease Control and Prevention, Guangzhou, Guangdong, People’s Republic of China; 7 Centre for Sami Research, Umea University, Umea, Sweden; 8 Fujian center for disease control and prevention, Fuzhou, People’s Republic of China; 9 School of Biological Sciences, University of Queensland, St Lucia, Australia; University of California, Davis, UNITED STATES

## Abstract

**Introduction:**

Dengue has become a more serious human health concern in China, with increased incidence and expanded outbreak regions. The knowledge of the cross-sectional and longitudinal epidemiological characteristics and the evolutionary dynamics of dengue in high-risk areas of China is limited.

**Methods:**

Records of dengue cases from 2013 to 2016 were obtained from the China Notifiable Disease Surveillance System. Full envelope gene sequences of dengue viruses detected from the high-risk areas of China were collected. Maximum Likelihood tree and haplotype network analyses were conducted to explore the phylogenetic relationship of viruses from high-risk areas of China.

**Results:**

A total of 56,520 cases was reported in China from 2013 to 2016. During this time, Yunnan, Guangdong and Fujian provinces were the high-risk areas. Imported cases occurred almost year-round, and were mainly introduced from Southeast Asia. The first indigenous case usually occurred in June to August, and the last one occurred before December in Yunnan and Fujian provinces but in December in Guangdong Province. Seven genotypes of DENV 1–3 were detected in the high-risk areas, with DENV 1-I the main genotype and DENV 2-Cosmopolitan the secondary one. The Maximum Likelihood trees show that almost all the indigenous viruses separated into different clusters. DENV 1-I viruses were found to be clustered in Guangdong Province, but not in Fujian and Yunnan, from 2013 to 2015. The ancestors of the Guangdong viruses in the cluster in 2013 and 2014 were most closely related to strains from Thailand or Singapore, and the Guangdong virus in 2015 was most closely related to the Guangdong virus of 2014. Based on closest phylogenetic relationships, viruses from Myanmar possibly initiated further indigenous cases in Yunnan, those from Indonesia in Fujian, while viruses from Thailand, Malaysia, Singapore and Indonesia were predominant in Guangdong Province.

**Conclusions:**

Dengue is still an imported disease in China, although some genotypes continued to circulate in successive years. Viral phylogenies based on the envelope gene suggested periodic introductions of dengue strains into China, primarily from Southeast Asia, with occasional sustained, multi-year transmission in some regions of China.

## Author summary

Dengue is the most prevalent and rapidly spreading mosquito-borne viral disease globally. Because of the multiple introductions, dengue outbreaks occurred in epidemic seasons in Southern China, supported by suitable weather conditions. Surveillance data from 2013 to 2016 in China showed that Guangdong, Yunnan and Fujian provinces were the high-risk areas, with dengue outbreaks occurring almost every year. However, knowledge has been lacking of the epidemiological characteristics and the evolution pattern of dengue virus in these high-risk areas. This study shows a variety of epidemiological characteristics and sources of imported cases among the high-risk areas in China, with likely origins primarily from countries in Southeast Asia. Seven genotypes of the DENV 1–3 variety co-circulated with DENV1-I, the main genotype, and DENV 2-Cosmopolitan, the secondary. Genetic relationships among viral strains suggest that the indigenous viruses in the high-risk areas arose from imported viruses and sometimes persisted between years into the next epidemic season, especially in Guangdong Province. Population movement has played a vital role in dengue epidemics in China. This information may be useful in dengue control, especially during epidemic seasons and in the development of an early warning system within the region, in collaboration with bordering countries.

## Introduction

Dengue is a mosquito-borne viral infectious disease caused by the four antigently distinct serotypes, DENV 1 through 4. Each serotype is classified into different genotypes based on complete E gene sequences [1]. Currently, dengue is endemic in more than 100 countries in many of the globe’s tropical and subtropical areas—predominantly in Southeast Asia, the Americas, and the Western Pacific, and less frequently in Africa and the Eastern Mediterranean WHO regions [2]. Because of the environmental changes, unprecedented population growth and movement, uncontrolled urbanization and spread of the mosquito vectors, the prevalence of dengue worldwide has increased 74.7% between 2006 and 2016 [3].

In China, no dengue was reported from 1949 to 1977. The first reported outbreak occurred in 1978 in Guangdong Province, which is located in Southeast China [4]. Over time dengue outbreaks have gradually expanded geographically from the Southern coastal regions of China to more Northern regions, including Fujian Province and Zhejiang Province, and to the more Westerly region of Yunnan Province [5]. Our previous study indicated that dengue was still an imported disease in China, and the outbreaks were probably caused by the imported cases [6]. However, the endemicity of dengue in China is still in dispute [7–10]. Guo *et al*., using the data from 2005 to 2011 argued for the endemicity of dengue infections in Guangdong Province, based on the facts of predominant circulation of DENV-1 for consecutive years and the co-existence of multiple serotypes since 2009 [7]. However, Sun *et al*. concluded that dengue was not endemic in Guangdong Province, although DENV 1–4 co-circulated [9]. There is no doubt that dengue has become more serious in recent years, with increased incidence and expanded outbreak regions [11]. For example, in 2014 an unprecedented dengue epidemic swept through Guangdong Province, affecting neighboring provinces as well: Fujian to the northeast and Guangxi to the west [12,13]. The incidence rate per one million reached as high as 34.6 in 2014, whereas it had ranged from 0.002 to 5.6 from 1990 to 2013 [11].

**Fig 1:**
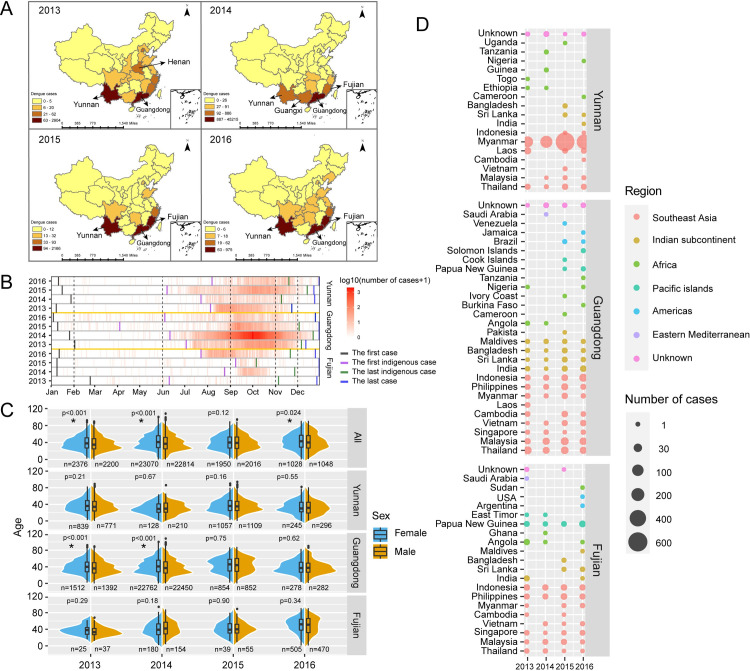
The epidemiology of dengue in high-risk areas of China from 2013 to 2016. A: The provinces having dengue outbreaks and the number of dengue cases at the province level (the map was downloaded from National Earth System Science Data Center, National Science & Technology Infrastructure of China (http://www.geodata.cn)). B: The heatmap of dengue cases in time series. The blue bar overlaps the green bar for Yunnan in 2013 and for Guangdong in 2016. C: Violin plot of the age distribution of dengue cases stratified by sex over a four-year period from 2013 to 2016. The top row shows the total cases and the rest the three provinces. Inside the violin plot is a box plot. The thick bar in the box represents the median, the thick black box in the center represents the interquartile range, and the thin black vertical line inside the violin plot represents the 95% confidence interval. D: The distribution and the number of imported dengue cases in the three high-risk provinces. The size of the dot represents the number of imported dengue cases, and the color represents the region from where the cases were imported. The unknown represents those cases recorded as imported but without exact places in the data source: China Notifiable Disease Surveillance System.

The space-time transmission pattern of dengue in China is changing. In the time dimension, dengue outbreaks occurred every year in some provinces in China [14]; in the space dimension, dengue outbreaks occurred simultaneously in different locations in the same year [15]. Fig 1A displays the status of dengue in China from 2013 to 2016.

No research has comprehensively studied the cross-sectional and longitudinal epidemiological characteristics and the evolutionary dynamics of dengue in high-risk areas of China. In this study, high-risk provinces are defined as those having outbreaks over two successive years. Individual case data recording demographic information and travel history can reflect the epidemiological characteristics. The genetic footprint in dengue virus sequences can reflect the molecular epidemiology and the evolutionary dynamics. Therefore, we have collected the individual dengue cases and dengue virus sequences from 2013 to 2016 to describe the epidemiology of dengue in high-risk areas of China, with the aim of exploring the origins of the epidemics. We hope that this study can provide more evidence on the debate of dengue’s endemicity in China.

## Methods

### Ethics statement

Ethical approval for the study was obtained from Qilu Hospital of Shandong University Ethical Committee (No. KYLL-2016(KS)-031).

### Data collection

Records of reported dengue cases from 2013 to 2016 were obtained from the China Notifiable Disease Surveillance System [16]. All cases are diagnosed according to the China National diagnostic criteria for dengue fever (WS216-2008), enacted by the National Health Commission of China. Information of dengue cases included age, gender, occupation, date of onset, type of diagnosis, and whether indigenous or imported, with source country. Routine case notification is performed by hospitals, as required by law. When dengue outbreaks occur in the community, possible dengue cases are also detected by active field investigation performed by health professionals. Therefore, dengue surveillance involves both passive and active case detection [17].

The criteria for imported cases included (1) residency or traveling experience in a dengue-endemic country or Taiwan of China and having been bitten by a mosquito within 15 days before symptoms appeared; or (2) the gene sequence of the virus isolated from the case being highly homologous with that reported by the country to/from which the patient had travelled [18]. An indigenous case is defined as the absence of evidence for the case being imported.

Dengue viruses detected in high-risk areas of China were recovered from samples of suspected dengue patients visiting hospitals from 2013 to 2016. Convenience sampling was adopted to amplify the E gene of DENV in the molecular epidemiology analysis. The E genes of 358 isolates were sequenced as described previously [19]. These sequences have been deposited in GenBank and assigned accession numbers (S1 Text).

### Epidemiological analysis

ArcGIS 10.1 was used to describe and plot the epidemiological characteristics in the spatial dimension. The distribution of the daily number of dengue cases was over-dispersed. To cover a large range of number of cases, including the days when there was no dengue case, a transformation was used: Log_10_ (number of dengue cases + 1). A heatmap was created to plot the time series of transformed dengue cases in the temporal dimension. Age distribution of dengue cases, stratified by sex, was summarized as violin plots, and the difference of age distribution between sexes was analyzed by a Wilcox test. R program (R core team, v3.5.1, Vienna, Austria) was used to plot these diagrams.

### Genotyping

The sequences collected from the high-risk areas of China were compared with published sequences by using the nucleotide blast program from the National Center for Biotechnology Information (NCBI). S1–S3 Tables were the references downloaded with the accession number, collection date and geographical region. All of the sequences were aligned using a program called *Multiple Alignment using Fast Fourier Transform* (MAFFT) [20]. Maximum Likelihood (ML) trees of each serotype were constructed using a General Time Reversible (GTR) substitution model of nucleotide evolution with gamma distribution and a heuristic subtree pruning and regrafting (SPR) branch-swapping search algorithm by FastTree 2.1 [21]. The Interactive Tree of Life program (iTOL) was used to visualize the annotated ML trees (https://itol.embl.de).

### Haplotype network analysis

The median joining network was used to visualize fine-scale genetic relationships based on single nucleotide polymorphisms in analyzed sequences. The network was constructed using DnaSP 6.12.3 [22] and PopART 1.7 [23].

## Results

### High-risk areas of dengue in China

From 2013 to 2016, a total of 56,520 dengue cases were reported in the China Notifiable Disease Surveillance System, with 4,779 in 2013, 45,212 in 2014, 4,238 in 2015 and 2,291 in 2016. The data showed that dengue outbreaks occurred in many provinces, with the highest number of cases in Guangdong Province, followed by Yunnan and Henan provinces in 2013; Guangdong, Guangxi, Fujian and Yunnan provinces in 2014; Guangdong, Yunnan and Fujian provinces in 2015; Fujian, Guangdong and Yunnan provinces in 2016. Yunnan and Guangdong provinces had dengue outbreaks in all the four data collection years, Fujian Province in the last three years. Therefore, Yunnan, Guangdong and Fujian provinces were defined as the high-risk provinces for dengue in China from 2013 to 2016 (Fig 1A).

### The epidemiology of dengue in high-risk areas of China

The heatmap in Fig 1B shows that dengue mainly occurred in August to November, although some variation does exist among different provinces. Imported cases occurred almost all year round, with the first imported cases mainly occurring in January and the last ones in December. The indigenous cases usually occurred with the first ones from June to August and the last from November to December. It is clear that Guangdong Province had the longest disease period with the highest number of cases, especially during the year of 2014, whereas Fujian had the shortest disease period and the lowest number of cases. Not only the outbreak duration but also the peak occurring time varied among these three high-risk provinces: in Guangdong the latter was mostly September–November, in Fujian in October in 2014 but shifted to August—September during 2016, and in Yunnan it was August—September in 2013 but shifted to October—November in 2015.

The violin plot in Fig 1C displays the age-year distribution stratified by sex for the three provinces and its sum. A total of 16 graphs is stacked together, with each row one location with their sum at the top, and each column one of the four years. The number of dengue cases in each graph is listed for each sex with a p-value to show the age comparison between male and female. The overall female to male ratio shows no significant female-male difference, 1.01:1. The age distribution of the total cases was significantly different between sexes in 2013, 2014 and 2016, with the median age a little younger in male than in female.

Based on the information of field epidemiology investigation, Fig 1D displays the regions from which dengue cases were imported, stratified by year and province. The result shows that dengue cases were predominantly imported from Southeast Asia (1,768 cases), but also from the Indian subcontinent (79 cases), Pacific islands (36 cases), Africa (31 cases), the Americas (7 cases), Eastern Mediterranean (3 cases) with some cases that could not be identified (Unknown). The dengue cases introduced to Yunnan Province was mainly from Myanmar (1,201 cases), and also from Laos (35 cases), Thailand (29 cases) and Malaysia (5 cases). The main source of dengue in Guangdong was Southeast Asia, with Thailand, Malaysia, Indonesia, Philippines, Vietnam, Myanmar, Singapore introducing infection each year. The Indian subcontinent was also an important source region, with India, Sri Lanka, Bangladesh, and the Maldives introducing some cases each year. Southeast Asia contributed the most imported cases to Fujian Province, with Indonesia, Philippines, Malaysia, Singapore, and Thailand introducing infection each year; in addition, Papua New Guinea was also an important imported country.

### Space-time dynamic transmission of dengue in China

**Fig 2:**
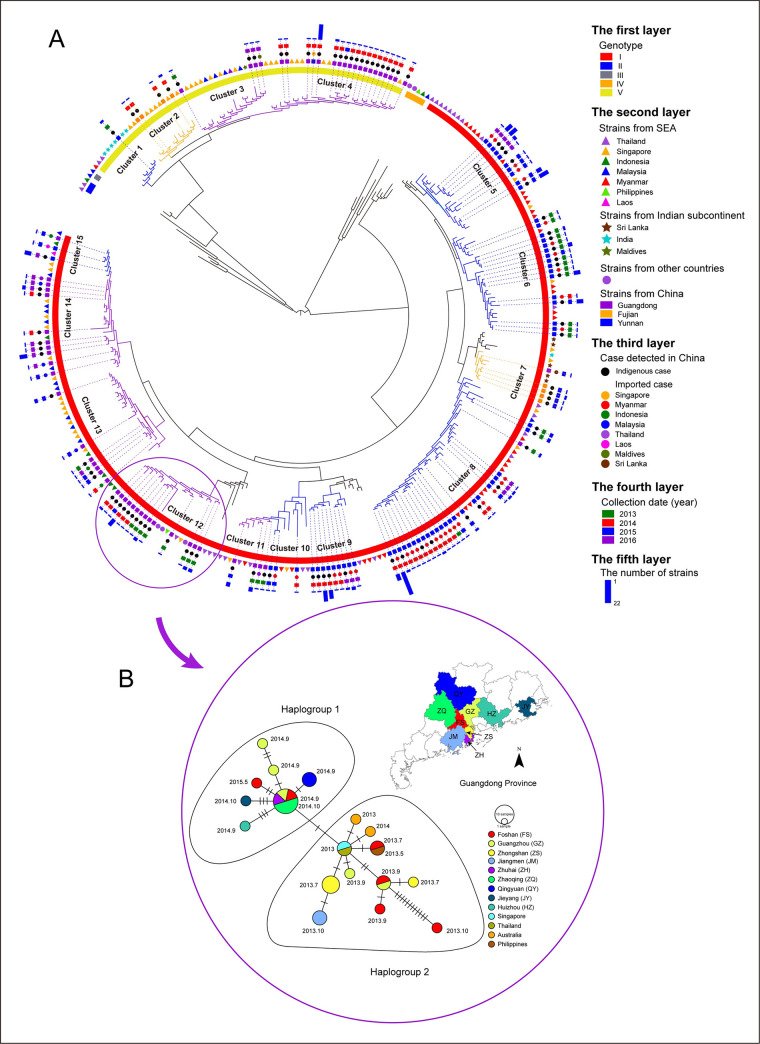
The phylogenetic relationship between dengue virus DENV 1 detected in the high-risk areas of China and the references downloaded from GenBank. A: The midpoint rooted ML tree of DENV 1, outside which there are five layers—see the figure legends. B: The fine-scale genetic relationships between DENV 1 detected in nine districts of Guangdong from 2013 to 2015, and those from Singapore, Thailand, Australia, and the Philippines, based on single nucleotide polymorphisms. Each notch on the links represents a mutated nucleotide site. The map was downloaded from National Earth System Science Data Center, National Science & Technology Infrastructure of China (http://www.geodata.cn).

**Fig 3:**
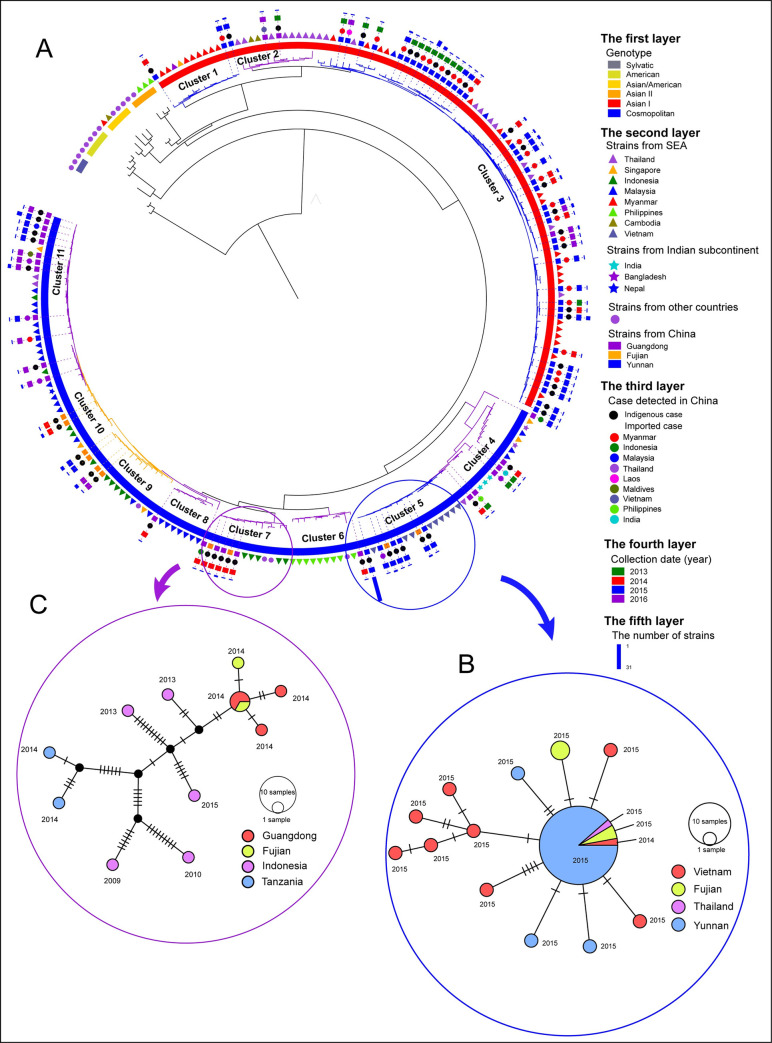
Same as Fig 2 but for DENV 2. A: The midpoint rooted ML tree of DENV 2, outside which there are five layers—see the figure legends. B: The fine-scale genetic relationships between DENV 2 detected in Yunnan and Fujian in 2015 and that from Vietnam and Thailand. C: The fine-scale genetic relationships between DENV 2 detected in Fujian and Guangdong in 2014 and that from Indonesia and Tanzania. Each notch on the links in B and C represents a mutated nucleotide site. The black node in C represents the inferred ancestral strain or a strain present in the population but not sampled.

**Fig 4:**
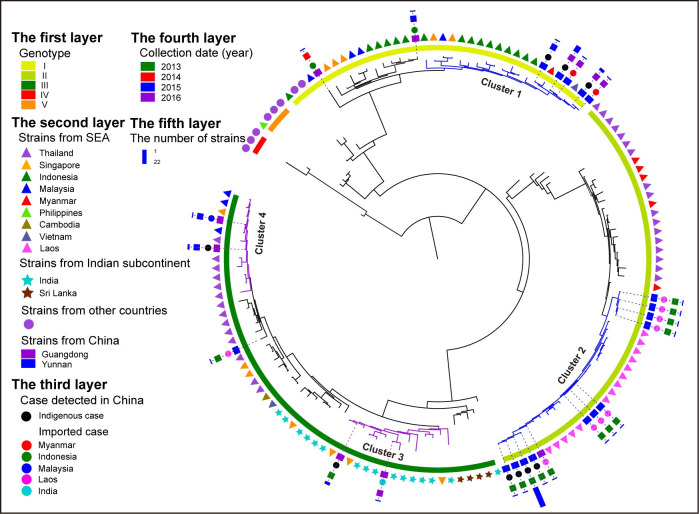
Same as Fig 2A but for DENV 3, without fine resolution added.

Figs 2–4 show the ML trees of DENV 1–3 constructed using the dengue viruses detected in the three high-risk provinces of China and reference strains from GenBank. The numbers of sequences in the ML trees were found to be 295 in DENV 1, 232 in DENV 2, and 142 in DENV 3. In the time dimension, Figs 2–4 show that almost all the indigenous cases in each province detected in different years separated into different clusters, with exception for Cluster 12 in Fig 2A, where indigenous cases in Guangdong formed one cluster for viruses DENV 1 detected from 2013 to 2015. Haplotype network analysis in Fig 2B shows that strains in Cluster 12 separated into two haplogroups for the Guangdong indigenous cases detected in different cities: from 2014 to 2015 in haplogroup 1 and in 2013 in haplogroup 2. Haplogroup 1 shows that the Guangdong strain detected in 2015 was the offspring of the Guangdong strain detected in 2014. The ancestor of haplogroup 1 and haplogroup 2 was the Thailand or Singapore strain detected in 2013 (Fig 2B).

In the space dimension, Figs 2A, 3A and 4 show that almost all the Yunnan, Guangdong and Fujian indigenous cases separated in different clusters, with exceptions for Cluster 5 and 7, as shown in Fig 3A. Cluster 5 in DENV 2 shows that the indigenous cases detected in 2015 in Yunnan and Fujian provinces clustered with Vietnam cases. Haplotype network analysis shows that 31 Yunnan indigenous cases, one Fujian indigenous case and one case imported from Thailand in 2015 have nucleotides identical with one Vietnam case detected in 2014. Other Vietnam cases and other indigenous cases in Yunnan and Fujian in Cluster 5 were the offspring (Fig 3B). Cluster 7 in DENV 2 showed that the Fujian and Guangdong indigenous cases detected in 2014 clustered with the Indonesia and Tanzania cases. Haplotype network analysis shows that two Guangdong indigenous cases and one Fujian indigenous case detected in 2014 have identical nucleotides. One other Guangdong and one other Fujian indigenous case detected in 2014 were the offspring (Fig 3C).

### Circulating dengue serotypes from 2013 to 2016

**Fig 5:**
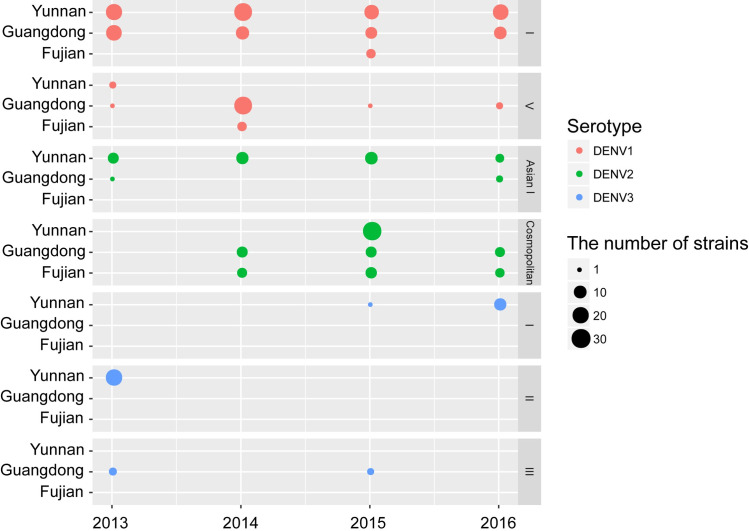
The molecular epidemiology of dengue viruses in the three high-risk provinces during 2013–2016. The color indicates different dengue virus serotypes and the size of dots represents the number of cases.

The genotypes of dengue viruses from indigenous cases of the three high-risk provinces are summarized in Fig 5, based on ML trees of DENV 1–3 during 2013–2016. Seven genotypes of DENV 1–3 were detected, with DENV 1-I (158 cases out of 357 total) as the main genotype and DENV 2-Cosmopolitan (76/357) the secondary. DENV 1-I, -V, DENV 2-Asian I, -Cosmopolitan, DENV 3-I, -II were detected in Yunnan Province, among which DENV 1-I and DENV 2-Asian I both were successively detected from 2013 to 2016. DENV 1-I, -V, DENV 2-Asian I, -Cosmopolitan, DENV 3-III were detected in Guangdong Province, among which DENV 1-I and -V both were also successively detected from 2013 to 2016 and DENV 2-Cosmopolitan successively detected from 2014 to 2016. DENV 1-I, -V and DENV 2-Cosmopolitan were detected in Fujian Province, among which DENV 2-Cosmopolitan was circulating from 2014 to 2016. DENV 1-I, -V, DENV 2-Asian I, -Cosmopolitan were concurrently circulating in two or three of the provinces in some years. The outbreak was caused by more than one genotype in Yunnan and Guangdong provinces in each of the four studied years. More than one genotype caused the outbreak in Fujian Province in 2014 and 2015.

## Discussion

In this study, we have focused dengue epidemiology on three provinces, Yunnan, Guangdong and Fujian, the high-risk areas of dengue in China from 2013 to 2016. Using the individual cases and virus sequences data from these provinces, we described the characteristics of dengue from field epidemiology and molecular epidemiology. The epidemic season was mainly from August to November. Imported cases occurred all year round and were mainly imported from Southeast Asia. This is consistent with the result of our phylogenetic analysis. Some genotypes caused outbreaks in successive years and circulated in more than one province in the same year but most viruses from the indigenous cases separated into different clusters. This indicates that the outbreaks were caused by repeated introductions in different years.

### Is dengue endemic in China?

Dengue has been detected in China since 1978, which is over 40 years. However, there is still controversy as to whether dengue is endemic in China [6–10]. Our results show that viruses from indigenous cases from Yunnan, Fujian, and most of Guangdong during 2013–2016 distributed discretely in different clusters in DENV 1–3 ML trees. They were clustered with viruses from Southeast Asia or the Indian subcontinent. This indicates that the outbreaks that occurred in different years were most likely to have been caused by the repeated introductions from Southeast Asia or the Indian subcontinent.

**Guangdong** Province was an exception. Although the viruses detected in Guangdong from 2013 to 2015 clustered with viruses from Singapore, Thailand, Australia and the Philippines, the haplotype network analysis shows that viruses in 2013 and 2014 were genetic descendants of viruses from Singapore or Thailand. However, the virus in Guangdong during 2015 was likely the genetic descendant of the virus in the same province in 2014. We see two possible explanations for the phylogenetic relationship between the Guangdong viruses of 2014 and 2015: 1) some viruses in 2014 survived to the next epidemic season and caused indigenous cases, and 2) the ancestor of the virus in 2015 was also the virus from Singapore or Thailand that was not sampled and is thus missed from the tree or network. We assumed that the first possibility resulted in the genetic pattern. However, we also noticed that the virus did not cause an outbreak in 2016, which means that the virus in Guangdong did not persist and evolve in situ. The same scenario was observed in Guangdong in 2002 and 2003, when dengue outbreaks successively occurred, and the same virus persisted for only two years and then disappeared with no in-situ evolution [6]. Even in Singapore, where dengue is hyper-endemic, some of the newly introduced dengue viruses spread within the country and established themselves into localized outbreaks most of which were short-lived [24]. Therefore, we believe that dengue is still an imported disease in China, although it has the possibility of occasionally surviving to the next epidemic season.

### Possible origin of imported cases to China

**Fig 6:**
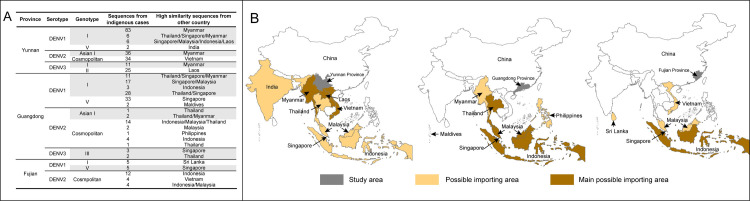
The possible origin of viruses detected from indigenous cases in the three high-risk provinces of China. A: The number of strains from indigenous cases in each of the three provinces, and the countries detecting high similarity of nucleotide. B: The possible importing countries. The map was downloaded from National Earth System Science Data Center, National Science & Technology Infrastructure of China (http://www.geodata.cn).

It should be noticed that the possible origins of indigenous cases in different high-risk areas of China is different, under the assumption that dengue is still an imported disease in China. Based on the results of ML trees of DENV 1–3 and the haplotype network analysis, the highly similar sequences from other countries with Yunnan, Guangdong and Fujian indigenous cases were summarized in Fig 6A and 6B. Southeast Asia and the Indian subcontinent were the possible imported sources, with Southeast Asia the main possible importing area. Specifically, in Yunnan Province Myanmar, Laos and Vietnam were the main possible importing areas, with Myanmar viruses causing the most indigenous cases. In Guangdong Province, Thailand, Malaysia, Singapore and Indonesia contributed most of the indigenous cases. In Fujian Province, it was Sri Lanka, Vietnam, Singapore, Malaysia and Indonesia, with Indonesia viruses causing the most indigenous cases.

### The role of human movement for dengue transmission

Given the restricted range of mosquito flying distance, human movement plays a critical role in dengue transmission. The growing scale of human mobility, particularly through airline travel, facilitates the co-circulation of dengue virus clusters [25]. One study indicated that the number of cases imported into China increased by 5.9% for every 10% increase in travel volume from dengue-endemic countries [26].

At a broad spatial scale (e.g., national, international), human movements may introduce or reintroduce dengue virus into a region with lower herd immunity [27]. The number of visitor arrivals in Yunnan Province has been steadily increasing from 1.5 million (1.0 million foreigners) in 2005 to 6.0 million (4.5 million foreigners) in 2016 [28]. The increase of human movement was accompanied by the increase of imported cases. The imported cases during 2005–2016 increased almost every year, and peaked in 2015. Of these imported cases, 42.9% were reported in Yunnan Province [29].

### The cases of Yunnan and Fujian provinces

**Yunnan** Province, which borders with Myanmar, Laos and Vietnam, had its first dengue outbreak in 2013 [19], followed by successive outbreaks in the next three years. Since the phylogenetic analysis during the four years shows viruses mainly clustered with the imported cases from Myanmar (Fig 2A and 3A), we contemplate that the outbreaks in Yunnan Province were probably attributed to repeated introductions from Myanmar. This is consistent with the field epidemiological investigation (Fig 1D). *Aedes aegypti* and *Aedes albopictus* are the primary and secondary vectors of dengue over the world. *Aedes albopictus* exists in nearly one third of China and has been the predominant vector of dengue in China [30]. In addition, a recent study showed that multiple-insecticide resistance was common in urban *Aedes albopictus* [31] which increases its survival. Furthermore, the study found that *Aedes aegypti* has expanded from Myanmar and Laos to China where it is found in all of the outbreak areas of Yunnan Province [32]. Therefore, both dengue vectors, *Aedes aegypti* and *Aedes albopictus*, co-colonized Yunnan Province. We know that dengue is transmitted more efficiently by *Aedes aegypti* than by *Aedes albopictus* [33]. Therefore, we believe that the contributing factors to the frequent dengue outbreaks in Yunnan Province may be 1) the persisting importation of dengue virus from adjacent areas of Southeast Asia, 2) the expanded distribution of *Aedes aegypti* from neighboring countries into Yunnan, and 3) the multiple-insecticide resistance in *Aedes albopictus*.

**Fujian** Province is located in the southeast coastal region of China. The first dengue outbreak there occurred in 1999, with 1,549 cases reported [34]. Subsequently, outbreaks occurred in 2004, 2007 and 2008 [35]. The frequency of dengue outbreak has increased since 2013. Three genotypes in DENV 1–2 caused the dengue outbreaks in Fujian province from 2014 to 2016. DENV 2-Cosmopolitan was successively detected in these three years. But its sources were different, as indicated by the DENV 2 ML tree, where viruses were discretely separated in different clusters (Fig 3A). In recent years, increasing global trade, labor exportation and population migration have facilitated the introduction of dengue virus from abroad. The number of foreign visitor arriving in Fujian province has increased from 0.5 million in 2000 to 2.5 million in 2016 [36]. In addition, Fujian province is also the home province of many overseas Chinese, especially from Southeast Asia. One study of dengue in Fujian province showed that imported cases had been reported every year from 2004 to 2014, with the number of imported cases from Southeast Asia increasing linearly with time [35]. Our study, by the field epidemiological investigation (Fig 1D) and the phylogenetic analysis (Fig 6), also confirms that the sources of dengue virus introductions were mainly from Southeast Asia.

### Interprovincial transmission?

The viruses detected in Fujian province in 2014 clustered with viruses detected in Guangdong Province and Indonesia (Cluster 7 in Fig 3), where one Fujian sequence and two Guangdong sequences have identical nucleotides. We see two possible explanations for this identity relationship: 1) the viruses were transmitted between these two provinces, 2) the viruses were from the same foreign country—Indonesia. Fujian is adjacent to Guangdong. Guangdong is called the commercial center of China for its economic development and there are more universities in Guangdong than in Fujian. During summer vacation (July to August), families in Fujian often travel to Guangdong for vacation. This is also the time when college students in Guangdong go back to Fujian. In addition, many people working in Guangdong travel back to Fujian to worship ancestors during the traditional Memorial Festival (about middle of August). Finally, an unprecedented outbreak occurred in Guangdong in 2014, with 45,215 cases reported. All of these influences increased the probability of introducing viruses to Fujian from Guangdong. The field epidemiological investigation also shows that some imported cases were from Guangdong. In the second possibility, the viruses from Indonesia are assumed to be introduced separately to these two provinces. However, the two possibilities are not mutually exclusive.

The existence of multiple channels of transmission—interprovincial and cross-border—can also explain why the viruses detected in Fujian in 2015 clustered with viruses detected in Yunnan Province, Vietnam and Thailand (Cluster 5 in Fig 3). Although we are not certain about interprovincial transmission of dengue virus, we can infer from this study that the origin of the viruses in the two clusters is Southeast Asia.

### Limitations

Although our study comprehensively describes the epidemiology of dengue in China from cross-sectional and longitudinal levels, the results should be interpreted cautiously, given its various limitations. First, there is currently no special reporting system for imported infectious diseases in China. The imported cases in this study were derived from the reports that the China Notifiable Disease Surveillance System received from the local CDC staff through field epidemiological investigation. It is possible that not all imported cases were identified. Second, the local CDC in China conducted convenience sampling to genotype the viruses during the dengue epidemics. Since there was no reference for the sample size, the sampling cannot sufficiently reflect the diversity of dengue virus in China. Third, the result on the possible origin of dengue virus in China is based on the similarity of nucleotides with strains from other countries. However, some countries have limited resources for virologic diagnostic and reporting capacity, which results in sequences missing from the GenBank, notably in Africa. Our study shows that some cases were imported from Africa, but molecular analysis shows that the imported cases did not cause indigenous cases in the study period.

Our data covers only a four-year period: 2013–2016, and only three provinces in China. Our study leaves the following question open: does local transmission continue through the “winter” season in other areas and in other years? In order to answer this question, future study is needed. Especially needed is more field epidemiology investigation, for example, monitoring dengue viruses from the dengue vectors, *Aedes aegypti* and *Aedes albopictus*. In addition, more dengue virus molecular surveillance is desirable. Above all, our study is a descriptive study; it is helpful to understanding the current dengue situation in China and to supporting health policies and practices for dengue prevention and control.

### Conclusion

In this study, we described the cross-sectional and longitudinal characteristics of dengue from field epidemiology and molecular epidemiology in Fujian, Guangdong, and Yunnan, the three high-risk provinces in China. Although 56,520 cases were reported in China from 2013 to 2016, we find that the dengue viruses have small possibility of surviving the winter to the next epidemic season. Therefore, this study supports the belief that dengue is still an imported disease in China. The sources of importation of dengue are distributed throughout tropical and subtropical areas, but Southeast Asia appears to be the main source of dengue viruses responsible for outbreaks in the high-risk provinces of China. Therefore, this study suggests that besides controlling mosquito density, population movement between Southeast Asia and China is an important factor in dengue outbreak control, especially during dengue epidemic seasons. Our results support the development of an early warning system within the region, in collaboration with bordering countries.

## Supporting information

S1 Text GenBank accession numbers. (DOCX)

S1 Table DENV 1 references. (XLSX)

S2 Table DENV 2 references. (XLSX)

S3 Table DENV 3 references. (XLSX)
