# Supplementary material for: The epidemiological characteristics of dengue in high-risk areas of China, 2013–2016
Source: PLoS Negl Trop Dis. 2021 Dec 20;15(12):e0009970. doi: 10.1371/journal.pntd.0009970 (PMC8687583; doi:10.1371/journal.pntd.0009970)
Supplement: S1 Text — (DOCX) [file pntd.0009970.s001.docx]

MW290474-MW290492, KU57103-KU570108, KU570132-KU570133, KU570127-KU570130, KF816158-KF816163, MW301582-MW301594, MW302763-MW302896, MW320621-MW320655, MW320656, MW332461-MW332465, MW332467-MW332471, MW332475-MW332479, MW332497-MW332501, MW332505-MW332509, MW332575-MW332592, MW332617-MW332618, MW332624-MW332625, MW332627, KF816147-KF816157, KF816164, KM651768-KM651782, KJ470712-KJ470716, KJ470718-KJ470763, KT453227, KT453242, KU291687, KU291689, KU291693, KU291694, KU291705, KU291708, KU291710, KU291711, KU291718, KU291719, KU291732, KU291733, KU291748-KU291751, KU291768, KU291769, KU291773-KU291780, KU291790, KU291804, KU291805, KU291811, KU291813,

KU291815, KU291816, MG737877, MG737884-MG737886, MG737912, MG737914, MG737937, MG737942, MG737963, MG737969, MG737994-MG737996, MG737998-MG738001, MG738046-MG738047, MG738052-MG738055, MG840587-MG840590, MN018338, MW404409, MW406479-MW406483
